# Determinants of the population growth of the West Nile virus mosquito vector *Culex pipiens* in a repeatedly affected area in Italy

**DOI:** 10.1186/1756-3305-7-26

**Published:** 2014-01-15

**Authors:** Paolo Mulatti, Heather M Ferguson, Lebana Bonfanti, Fabrizio Montarsi, Gioia Capelli, Stefano Marangon

**Affiliations:** 1 Istituto Zooprofilattico Sperimentale delle Venezie, Viale dell’Università 10, 35020 Legnaro (Padua), Italy; 2 University of Glasgow, Institute of Biodiversity, Animal Health and Comparative Medicine, Glasgow G12 8QQ Scotland (UK

**Keywords:** *Culex pipiens*, Culicidae, Mosquito population dynamics, Density dependence, West nile virus, Disease vectors

## Abstract

**Background:**

The recent spread of West Nile Virus in temperate countries has raised concern. Predicting the likelihood of transmission is crucial to ascertain the threat to Public and Veterinary Health. However, accurate models of West Nile Virus (WNV) expansion in Europe may be hampered by limited understanding of the population dynamics of their primary mosquito vectors and their response to environmental changes.

**Methods:**

We used data collected in north-eastern Italy (2009–2011) to analyze the determinants of the population growth rate of the primary WNV vector *Culex pipiens*. A series of alternative growth models were fitted to longitudinal data on mosquito abundance to evaluate the strength of evidence for regulation by intrinsic density-dependent and/or extrinsic environmental factors. Model-averaging algorithms were then used to estimate the relative importance of intrinsic and extrinsic variables in describing the variations of per-capita growth rates.

**Results:**

Results indicate a much greater contribution of density-dependence in regulating vector population growth rates than of any environmental factor on its own. Analysis of an average model of *Cx. pipiens* growth revealed that the most significant predictors of their population dynamics was the length of daylight, estimated population size and temperature conditions in the 15 day period prior to sampling. Other extrinsic variables (including measures of precipitation, number of rainy days, and humidity) had only a minor influence on *Cx. pipiens* growth rates.

**Conclusions:**

These results indicate the need to incorporate density dependence in combination with key environmental factors for robust prediction of *Cx. pipiens* population expansion and WNV transmission risk. We hypothesize that detailed analysis of the determinants of mosquito vector growth rate as conducted here can help identify when and where an increase in vector population size and associated WNV transmission risk should be expected.

## Background

In the last decade, changes in global climate have raised concern as being a potential trigger for the expansion of infectious diseases, and in particular those borne by arthropod vectors (Vector Borne Diseases – VBDs), into previously unaffected areas [1]. The emergence and recurrence of VBDs such as Blue Tongue, West Nile Disease, and Chikungunya into areas of Europe formerly thought not to be environmentally suitable for their transmission have been considered as a confirmation of this threat within temperate zones [2-5]. Nevertheless, climate change alone may not represent a sufficient cause to explain the behaviour or the expansion of VBDs in newly affected areas [6]. Other factors, including socio-economic and land-use factors, may nullify or counter any impacts of climate change in enhancing conditions for VBD establishment and transmission (e.g. as has been hypothesized for malaria in Camargue and in south-eastern England [6]). However, other areas have reported an expansion of VBDs in areas where environmental conditions were not thought to favour transmission, such as West Nile in USA [6]. The variable response of VBD transmission to environmental change [6,7] indicates that prediction of the risk of emergence and spread into new areas is complex, and likely requires detailed knowledge of the specific pathogen-vector system and their environmental dependencies. We hypothesize that a thorough understanding of the core ecological determinants of the population dynamics of arthropod vectors is fundamentally required to improve predictions of the potential extent and rate of VBD expansion and risk.

West Nile virus (WNV) has become one of the most widely distributed arboviruses in the world [8]. The virus is normally maintained and spread through a bird-mosquito cycle involving ornithophilic *Culex* spp. mosquitoes [8,9]. However, the virus can also be spread to a wide range of incidental hosts including humans and horses, via mammophilic and/or anthropophilic mosquito species (including *Aedes* spp. and *Ochlerotatus* spp.), and it has the potential to cause severe illness characterised by neurological disorders [10]. The presence of WNV has also been sporadically recorded in hard (*Ixodus* spp. and *Amblyomma* spp.) and soft ticks (*Argas* spp.), with some cases of transmission to other hosts [9]. Various non-vectorial modes of transmission have also been observed in a variety of mammals, including humans, suggesting the possibility that a WNV cycle could be sustained in the absence of mosquito vectors [11]. Nevertheless, non-vectorial routes, and also non-mosquito vectors, appear to play a very minor role in the maintenance of the WNV cycle in nature [12].

West Nile Virus has been circulating in Europe for more than 30 years, primarily in central and Mediterranean countries, with cases being recurrently reported both in humans and in horses [13]. The first appearance of WNV in Italy occurred in 1998 in Tuscany, with 14 infected horses and no human cases [14]. No further clinical manifestations of WNV were reported until 2008, when 251 horse premises tested positive for WNV in the Po Valley of north-eastern Italy [15]. Since then WNV has been recurrently detected in horses, wild birds, and humans [16,17], suggesting the likely endemisation of the disease [18]. In 2010, WNV was also identified in *Culex pipiens* mosquitoes in the neighbouring regions of Veneto and Emilia Romagna. Since then the virus has been isolated from *Cx. pipiens* only, suggesting these mosquitoes are the primary WNV vectors in Italy [19].

Despite the widespread distribution of *Cx. pipiens,* understanding of the determinants of their population dynamics both within Italian WNV transmission areas, and other parts of Europe is rather limited. Previously it has been assumed that environmental factors such as temperature and rainfall are the overwhelming drivers of mosquito population growth [20]. However, recent investigations of other tropical and temperate mosquito species indicate that endogenous density dependence may have a strong influence on their population growth, as arising primarily from intra-specific competition for resources within their aquatic larval habitats [21-23]. The primary implication of this phenomenon is that intra-specific competition may limit the size of vector populations at levels below which would otherwise be expected from abiotic conditions alone. As such, existence of strong density-dependent regulation within vector populations may limit the potential for WNV transmission to below that estimated from environmental conditions on their own.

Here the existence of such a phenomenon within the putative main WNV vector in Italy was investigated for the first time. Using longitudinal data obtained over three years from a WNV surveillance programme implemented in north-eastern Italy, the seasonal abundance of *Cx. pipiens* in north-eastern Italy was analyzed to quantify the relative contribution of density dependence and exogenous environmental factors to their population growth. This information will provide a more realistic understanding of the constraints on WNV vector populations within Europe, and facilitate prediction of their spatial and/or temporal expansion and associated epidemiological impact. Ultimately, such detailed knowledge of vector population determinants could provide a more reliable framework for development of risk-based WNV surveillance and early detection programs.

## Methods

### Mosquito collection

**Figure 1:**
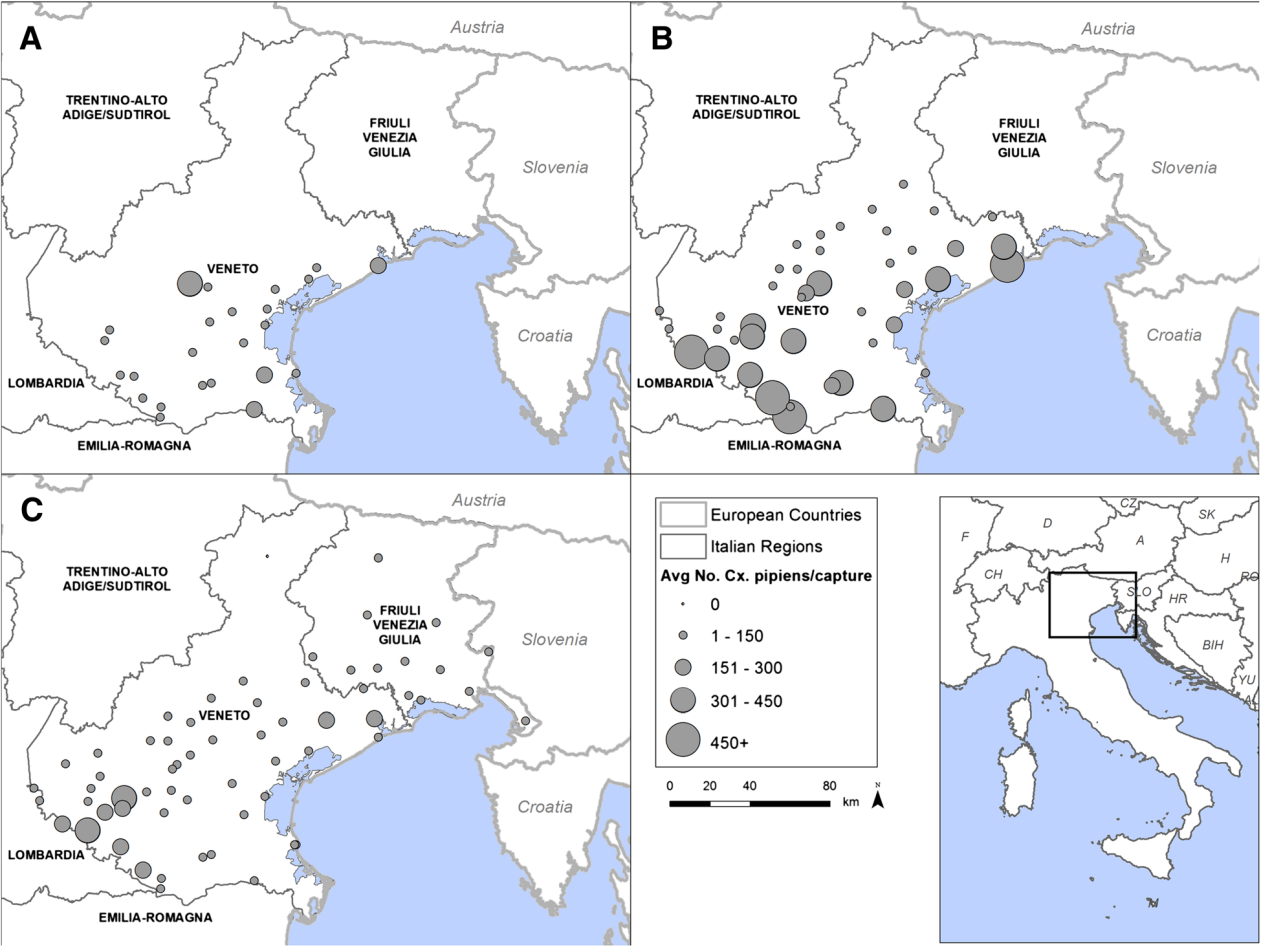
**Geographical distribution of *Cx. pipiens *in the study regions.** Average number of mosquitoes per capture night during the WNV entomologic surveillance period. **A**. 2009, **B**. 2010, **C**. 2011.

Mosquitoes were captured in the context of the WNV entomological surveillance plan in Veneto and Friuli Venezia Giulia (FVG) regions, north-eastern Italy. The surveillance activities ran between 2009 and 2010 in Veneto only, and in 2011 in both Veneto and FVG. Sampling sites were located in a variety of settings including livestock farms, protected natural areas, and at private houses. The number of sites surveyed each year varied in accordance to the routine surveillance programme operating during the study period (Figure 1). At each site mosquito collection was conducted using CDC traps baited with carbon dioxide (IMT® – Italian Mosquito Trap), which predominantly capture host-seeking females [24]. In each year, one overnight sample was conducted at each site every 15 days from the first week of May until two consecutive negative mosquito captures (usually occurring between the end of October and first week of November), which was assumed to indicate the end of the mosquito season.

Only *Cx. pipiens* has been considered in the analyses, since it has been acknowledged as being the most important WNV vector in Italy [19]. Moreover, as the two subspecies belonging to *Cx. pipiens* (*Cx. pipiens pipiens* and *Cx. pipiens molestus*) are morphologically indistinguishable, the analyses were conducted on *Cx pipiens* complex as a whole.

### Environmental monitoring

Environmental data were recorded at weather stations located in the closest proximity to mosquito traps (mean distance: 5,095 m, 95% CI 4,507 – 5,683 m). Collected data included daily measurements of the maximum, minimum and mean temperatures, minimum, maximum, and average relative humidity rates, and cumulative daily precipitation.

Climatic data were further processed to obtain summary measurements of environmental conditions during mosquito larval development. On the basis of the known duration of the *Cx. pipiens* larval development period under field condition [25,26], we assumed environmental conditions throughout the 15 day period prior to the day of adult mosquito capture to be a suitable representation of conditions during their larval development. The cumulative precipitation and the average relative humidity in the 15 days preceding each capture were estimated, and the total number of rainy days during this period was calculated as the sum of days with precipitation higher than 1 mm [27]. The impact of temperature during larval development was estimated by computing the total Growing Degree Days (GDD) during the 15 days prior to adult collection. The GDD indicates the exposure to temperatures that may affect the development of an organism [28]. It has been extensively used in agrometeorology to predict plant and pest development, but also in medical entomology [28,29]. The computation of GDD is based on the definition of temperature thresholds, above and below which the organism cannot develop and its activities are limited. For each sampling day, the GDD was calculated as following:

$$\mathrm{GDD}=\;\left\{\begin{array}{c} \;0\;\mathrm{if}\;T_{\text{mean}}<L.T.\\ \;T_{\text{mean}}-L.T.\;\mathrm{if}\;L.T.<T_{\text{mean}}\\ \;U.T.\;\mathrm{if}\;T_{\text{mean}}>U.T. \end{array}\right)\;<U.T.;$$

where *T*_*mean*_ is the daily average temperature, and *L.T.* and *U.T.* are the lower and upper temperature thresholds respectively. In the present study, values of 13°C and 33°C were used for these lower and upper thresholds respectively [30,31]. The daily GDDs were then summed to obtain the cumulative GDD for the 15 days preceding the mosquito capture.

*Culex pipiens* mosquitoes can undergo a diapause, defined as a period when development and activity is suppressed [32]. Entrance into the diapause stage is strongly dependent on photoperiod, with the proportion of mosquitoes in diapause rapidly decreasing as the length of daylight increases [32]. Consequently the length of daylight was recorded for each capture day to test its association with mosquito population dynamics (data collected from http://aa.usno.navy.mil/).

A summary of the environmental variables included in the analyses is provided in Table 1.

**Table 1:** Environmental variables used in the generalized linear mixed-effects models

**Variable**	**Description**
PREC	Daily cumulative precipitation (mm)
GDD	Growing degree days
HMN	Daily minimum relative humidity (%)
PREC.15d	Cumulative precipitation in 15 days prior to capture(mm)
DPREC.15d	Number of rainy days in 15 days prior to capture
HAV.15d	Average relative humidity in 15 days prior to capture (%)
GDD.15d	Growing degree days in 15 days prior to capture
LDM	Length of daylight (minutes)

### Statistical analyses

Estimates of mosquito abundance based on single trapping events were processed to obtain estimates of the population growth rate of *Cx. pipiens* at each site. To reduce the effect of short term fluctuations in the number of mosquitoes captured against the longer-term background trend, the raw number of mosquitoes per capture was smoothed using a centered rolling computed on three adjacent captures. Furthermore, extreme values of mosquito abundances were removed, by excluding values greater than the 99^th^ percentile from the data set. The per-capita growth rate of *Cx. pipiens* at each sampling occasion was computed as:

$$r_{t}=\ln\frac{N_{t+1}}{N_{t}};$$

where *N*_*t*_ is the population density at time *t*, *N*_*t+1*_ is the mosquito abundance at the next capture.

A series of population growth models were fitted to evaluate the strength of evidence for regulation by density-dependent and independent environmental factors (concurrent or time-lagged). In the first step of analysis, a reference model of mosquito population dynamics was constructed that included data on the length of photoperiod (LDM) at capture. Given *Cx. pipiens* reproductive diapause is known to be highly dependent on photoperiod (which varied only temporally but not spatially throughout the study zone), LDM was incorporated into the reference model as a means of controlling the considerable variation it generates in mosquito growth rates (LDM, Table 1). By incorporating LDM into the reference model, the impacts of the additional environmental and density dependent factors under study could be more precisely distinguished from background variation due to photoperiodicity. A set of 15 alternative statistical models were fit to investigate the effect of adding single extrinsic or density-dependent variables to the reference model. Density independent models included the reference model, an exponential growth model, and models in which only the impact of individual environmental variables were evaluated. Ricker-logistic and Gompertz-logistic models were used as baseline models of density-dependent population growth. Additional density-dependent models were tested by considering known environmental correlates of mosquito habitat as potential regulators of the carrying capacity [22], as follows:

$$r_{\text{Ricker}}=r_{m}\left(1-\frac{N}{K\times \mathrm{Env}}\right);$$

And

$$r_{\text{Gompertz}}=r_{m}\left(1-\frac{\ln N}{\ln\left(K\times \mathrm{Env}\right)}\right);$$

where, *r*_*m*_ is the maximum intrinsic growth rate, assumed in our models to be equal to the highest observed growth rate per capture site over the whole observation period, *N* is the abundance of mosquitoes per capture, *K* is the carrying capacity (which was assumed to vary for the three study years, and was set as the maximum number of mosquitoes recorded per capture site in each year of observation), and *Env* is the environmental variable assumed to be a proxy of critical habitat. We assumed that environmental factors likely related to the availability of suitable larval habitat would have the strongest effects on determining carrying capacity. Therefore, we considered the average humidity and cumulative precipitation in the 15 days prior to adult capture as the environmental factors most likely to modify the carrying capacity of mosquito populations [33,34].

All of the statistical models were fitted using Maximum Likelihood mixed-effects linear regression (Generalized Linear Mixed-effects Model, GLMM) [35]. Grouping variables related to the year of observation (2009, 2010 or 2011) and site of capture were considered as crossed random effects. For all of the 15 alternative models the Akaike’s information criterion corrected for small sample size was calculated, and the strength of support was evaluated through calculation of their Akaike weights (*w*AIC_c_ ), which may be interpreted as the probability that a model is the most likely [36]. For each of the tested GLMMs, a likelihood-ratio based pseudo-R^2^ (*R*^*2*^_*LR*_) was calculated as a measure of goodness of fit [37,38]. The model with the strongest degree of statistical support was selected as the most appropriate base model for further investigations of the role of additional environmental factors on mosquito populations.

After the best base model for population growth was identified, additional model selection procedures were conducted to assess the further contribution of other environmental factors. Model selection was based on Information Theoretic methodologies based on the corrected Akaike Information Criterion (IT-AIC_c_) [39-41]. A full initial model was created by including all of the environmental variables considered (Table 1), and a set of models containing the terms included in base population model and subsets of terms of the global model was generated. All of the models were compared through *w*AIC_c_ to assess whether there was a single model with high statistical support, or many models with similar values of *w*AIC_c_. In the case of uncertainty in model selection, a model averaging algorithm was conducted which allowed combination of the parameter estimates from a selected set of models, considering the contribution of each model as proportional to its likelihood weight [39,41]. The set of models whose total cumulative *w*AIC_c_ was at least equal to 0.95 were selected for model averaging. In doing this, it can be inferred that the selected set of models is expected to contain the AIC-best model with a probability of 0.95 if further data were added [41]. The importance of each single variable in the model with averaged coefficients, were calculated as the cumulative *w*AIC_c_ for the models in which the variables were included as predictors. This value, may also be interpreted as the probability that a particular variable is included in the best AIC_c_ model [41].

The generalized mixed-effect models were fitted using the *nlme* package [42], the IT-AIC_c_ approach was performed through the package *MuMIn*, in the R statistical software version 2.15.1 [43].

## Results

### Annual trends in mosquito distribution and abundance

**Figure 2:**
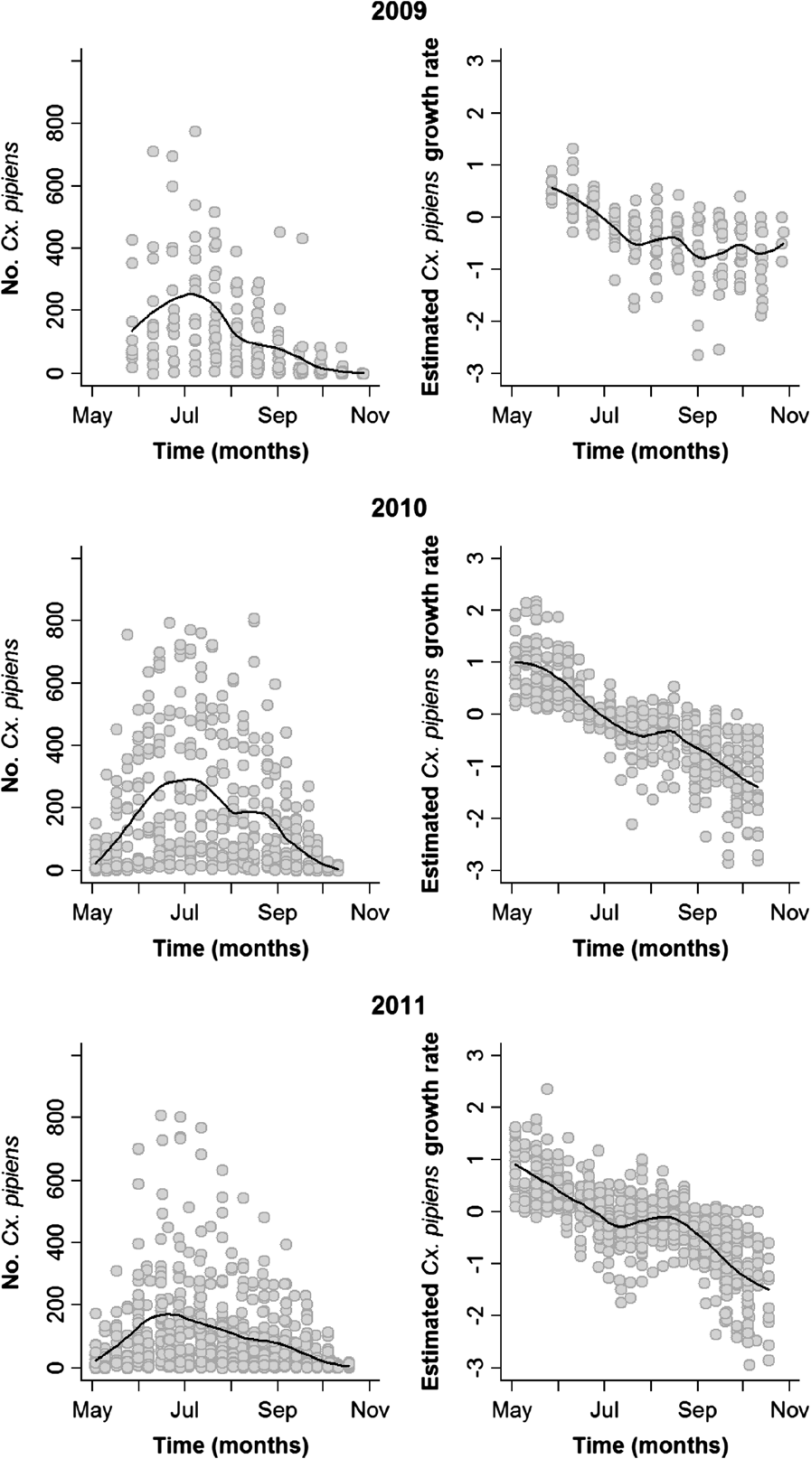
**Overall abundance of *Cx. pipiens *per night, and growth rates during the observation period.** Each gray dot represents a single observation per capture site/night.

The duration and number of captures during the three study years are summarized in Table 2. The average number of mosquitoes captured per trap night varied significantly between years (F_2, 1496_ = 21.02, p < 0.001). *Culex pipiens* was the dominant species within the sampled mosquito community (>78% of all mosquitoes sampled, in all years, Table 2). This species was also the most widespread, being sampled at all sites in all years except for one high altitude (980 m) location in 2011 (Figure 1). The average abundance of *Cx. pipiens* varied significantly between years (F_(2, 1471)_ = 21.33, p < 0.001), with post-hoc Tukey’s comparisons revealing that its abundance was greater in 2010 than in other years (p = 0.002 for comparison with 2009; p < 0.001 for comparison with 2011). *Culex pipiens* densities recorded in 2009 and 2011 were not significantly different (p = 0.47; Table 2). The abundance of *Cx. pipiens* followed a similar seasonal pattern in all three observation years, with numbers peaking at the end of June and start of July (Figure 2). This corresponded to a peak in population growth rate at the beginning of the surveillance season (between the end of May and mid-June) which then decreased constantly until the end of the observation period (Figure 2).

**Table 2:** Number of mosquitoes collected during the sampling periods 2009-2011

	**2009**	**2010**	2011*
Sampling period (first capture – last capture)	27 May – 11 Nov	3 May – 26 Oct	3 May – 25 Oct
Total collected mosquitoes	35, 129	137, 897	85, 136
Total number of captures	222	536	733
Avg. no. mosquitoes per capture	158. 24 (S.E.: 17. 77)	257. 27 (S.E.: 24. 64)	116. 14 (S.E.: 7.85)
Total collected *Cx. pipiens*	27, 721 (78. 91%)	119, 847 (86. 91%)	68, 932 (80. 97%)
Avg. no. *Cx. pipiens* per capture	124.87 (S.E.: 15. 01)	223. 60 (S.E.: 22. 40)	94. 04 (S.E.: 6. 74)
Total no. of identified species	12	16	14

The table report results of the capture activities across all study sites; the mean mosquito abundance per capture, and density of *Cx. pipiens* are also reported.

*Including both Veneto and Friuli Venezia Giulia.

### Predictors of mosquito population growth rate

Preliminary analyses indicated that the density-independent models provided the poorest description of *Cx. pipiens* seasonal population growth (Table 3). Moreover, several of the environmental variables taken individually provided no better fit to population growth dynamics than a simple random walk model, indicating that they were of limited relevance to mosquito population growth. Only growing degrees-day (both concurrent to the mosquito captures and the 15-days cumulative measure), and the number of rainy days and cumulative precipitation in the 15 days prior to mosquito collections added significant information to better describe the growth rate dynamics (Log Likelihood Ratio Tests: GDD vs Random Walk, χ_1_^2^ = 26.84, p < 0.001; GDD in 15 days vs Random Walk, χ_1_^2^ = 151.60, p < 0.001; Precipitation in 15 days vs Random Walk, χ_1_^2^ = 3.84, p = 0.05; Number of Rainy Days in 15 days vs Random Walk, χ_1_^2^ = 5.25, p = 0.021).

**Table 3:** **Statistical models built to define the baseline population dynamics of** ***Cx. pipiens***

**Model**	**ΔAIC**_**c**_	***w*** **AIC**_**c**_	***R***^***2***^_***LR***_
Gompertz	0.00	> 0.999	0.59
Gompertz mod. by humidity	33.26	< 0.001	0.58
Ricker	192.99	< 0.001	0.52
Ricker mod. by humidity	195.03	< 0.001	0.52
Growing degree-day (15 dd)	209.23	< 0.001	0.52
Growing degree-day	334.21	< 0.001	0.47
Gompertz mod. by by precipitation	354.26	< 0.001	0.46
Rainy days (15 dd)	355.79	< 0.001	0.46
Precipitation (15 dd)	357.21	< 0.001	0.46
**Reference model**	**359.02**	**< 0.001**	**0.46**
Exponential	359.02	< 0.001	0.46
Ricker mod. by precipitation	360.05	< 0.001	0.46
Minimum daily humidity	360.62	< 0.001	0.46
Precipitation	360.77	< 0.001	0.46
Average daily humidity (15 dd)	361.02	< 0.001	0.46

The models are reported in order of increasing ΔAIC_c_, the reference model (i.e. mode with only length of daylight as explanatory variable) is reported in boldface.

Both Ricker- and Gompertz-logistic models performed better than any single density-independent environmental factor. Within alternative density-dependent models, those in which the mosquito carrying capacity was modified by proxy indices of habitat availability (i.e. average humidity and cumulative precipitation in the 15 days prior to mosquito capture), had lower explanatory power than base Ricker- and Gompertz-logistic alternatives (Table 3). Overall, the Gompertz-logistic model provided a substantially better representation of the seasonal growth dynamics of *Cx. pipiens* populations than any of the other base models considered, with an overwhelming *w*AIC_c_ support greater than 0.999 (Table 3). Only 6 of the 15 alternative growth models resulted explaining more variation than the reference model: the base Gompertz- and Ricker-logistic models (*R*^*2*^_*LR*_ = 0.59, and *R*^*2*^_*LR*_ = 0.55, respectively), their version with carrying capacity modified by humidity (*R*^*2*^_*LR*_ =0.58 and *R*^*2*^_*LR*_ = 0.55, for the modified Gompertz and Ricker models respectively), and the density-independent models with GDD and the cumulative GDD in 15 days as predictor (*R*^*2*^_*LR*_ *=* 0.47, and *R*^*2*^_*LR*_ = 0.52, respectively).

A total of 128 models were built adding all of the possible combination of variables to the best base model, one combination at a time (Additional file 1: Table S1). The comparison of the models did not allow us to define a single best model, as the resultant overall statistical support was very low (Additional file 1: Table S1). The highest ranked model had a *w*AIC_c_ of 0.077, and 48 models were required to reach a cumulative *w*AIC_c_ of 0.95. The goodness of fit did not vary substantially between the models (minimum *R*^*2*^_*LR*_ = 0.59, maximum *R*^*2*^_*LR*_ = 0.60) (Additional file 1: Table S1). Nevertheless, the IT approach applied allowed definition of the relative importance of every environmental variable in the whole set of models, by indicating the cumulative *w*AIC_c_ of the models in which they were included (Table 4). The model with averaged parameters showed that only a few factors had significant effects on the growth rates: the length of the photoperiod, the density-dependent factor, and the cumulative Growing degrees-day in the 15 days prior to the capture (Table 4).

**Table 4:** **Coefficients of the fixed effects for average model for *Cx. pipiens *** **population dynamics**

**Coefficient**	**Estimate**	**95% C.I.**	**S.E.**	**Importance**
(Intercept)	-0.234	-0.270; -0.197	0.019	--
LDM	0.789	0.740; 0.839	0.025	1.00
Ln(N)/Ln(K)	-0.421	-0.471; -0.371	0.026	1.00
GDD	0.029	-0.007; 0.064	0.018	0.57
GDD.15d	-0.106	-0.151; -0.061	0.023	1.00
PREC	0.004	-0.023; 0.031	0.014	0.25
PREC.15d	0.002	-0.032; 0.036	0.017	0.26
DPREC.15d	-0.021	-0.055; 0.013	0.018	0.42
HMN	0.000	-0.028; 0.028	0.014	0.24
HAV.15d	-0.014	-0.050; 0.021	0.018	0.32

The importance of each variable provides information on the cumulative *w*AIC_c_ of the models that include the term.

Comparisons between the relative effects of the different factors were facilitated by scaling and centering the environmental variables before model fitting. Therefore, it was possible to make inferences simply considering the magnitude of coefficients, with higher absolute values indicating stronger effects on the population growth rates [44], despite the environmental factors being recorded with different units of measure. The highest absolute value for the coefficients was associated with the length of daylight. This association with daylight is hypothesized to be driven by the known influence that the shortening of photoperiod has on determining the beginning of the diapause phase in *Cx. pipiens*[32]. The positive sign of the coefficient indicates that mosquito reproduction capacity was higher when daylight lasted longer (i.e. in summer), while it dropped with a decrease of the photoperiod. The form of density-dependence acting on *Cx. pipiens* was predicted to be strongly negative, indicating that when the population density approaches the carrying capacity, their growth rate decreases. The impact of the cumulative GDD was also found to be negative (Table 4), which could be indicative of negative effects occurring over prolonged periods where temperatures occur near the maximum tolerance for *Cx. pipiens*, and/or may have been related to limited ability of larvae to acclimate above specific temperature thresholds (e.g. 25°C for *Cx. pipiens*) [45]. Alternatively, this apparently negative impact of GDD may be a product of its correlation with another environmental variable not measured here that has a directly negative association on GDD (e.g. predator abundance, etc). Further research into this potential relationship with GDD is required to clarify its impact on mosquito population growth.

Overall, the combination of length of daylight, density-dependence, and cumulative GDD in the 15 days prior to capture generated the best description of *Cx. pipiens* growth dynamics. All three of these variables were included in the top 48 models (out of a set of 128) that accounted for a cumulative *w*AIC_c_ ≤0.95. In contrast, the other environmental factors considered (daily GDD, daily precipitation, cumulative precipitation and number of rainy days within 15 days prior to mosquito collections, and minimum daily humidity and average humidity in 15 days before mosquito captures) were of relatively low significance (Table 4), as interpreted from their lower probability of being included in the best AIC_c_ model [41].

**Figure 3:**
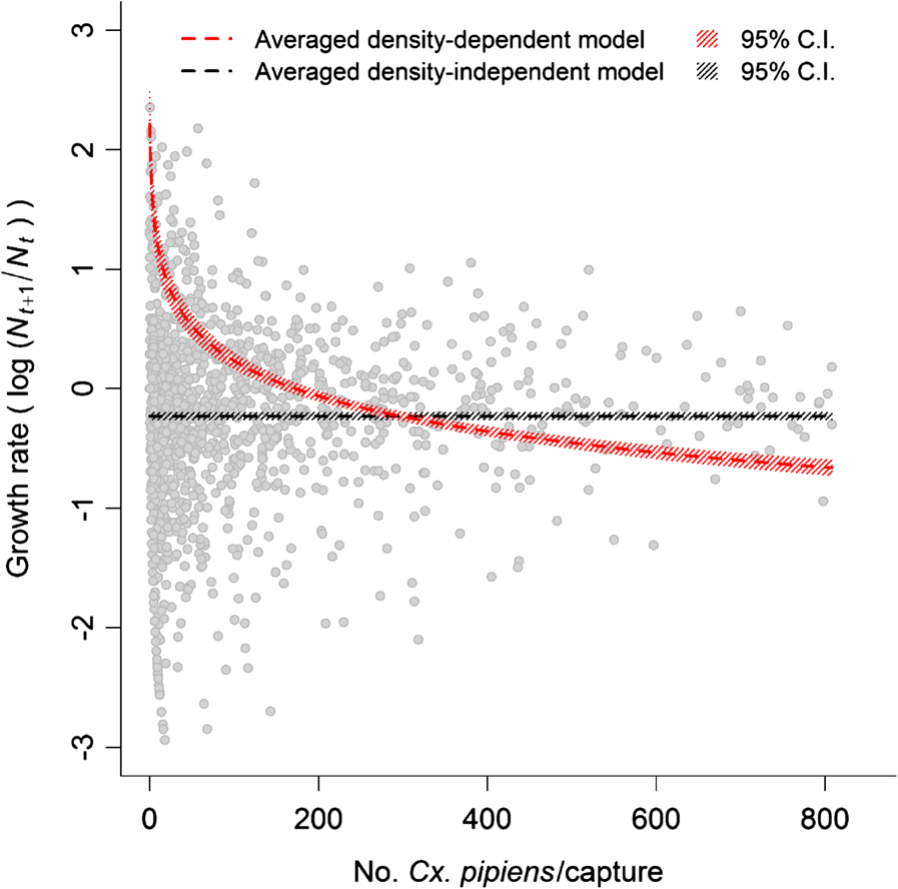
**Comparisons between the different *Cx. pipiens *growth dynamic models.** Each gray dot represents the observed per-capita growth rate.

The effect of mosquito density on the population dynamics was also visualised by plotting the predicted growth rates resulting from the averaged full model with density dependent factors, and the prediction obtained from an averaged model in which density-dependence was not included (Figure 3). While the density-independent model showed a constant growth rate through the whole range of mosquito densities, the model based on the Gompertz-logistic population growth predicted decreasing growth rates as mosquito abundance increased (Figure 3).

## Discussion

West Nile Virus mosquito vector species ecology and dynamics are commonly considered to depend mostly on external environmental factors, such as rainfall or temperature [20,46,47]. While all mosquito populations are likely regulated by such environmental factors to some extent, recent evidence from tropical species indicates that density-dependence can also play a large role in determining population growth rates [21,22]. The existence of density-dependence has not been widely investigated in temperate mosquito species, nor in vectors of WNV, therefore, we developed and tested a series of statistical models, to investigate the relative importance of density-dependent versus independent factors in predicting the population growth rates of the WNV vector *Cx. pipiens*, in a recurrently affected area of Italy.

Our analyses showed that density-dependent models provide a much better description of *Cx. pipiens* growth rates during the study period than any extrinsic environmental variable on its own. This suggests that mosquito density has a more significant effect on regulating the dynamics of *Cx. pipiens* than previously thought. Moreover, little or no interactions between environmental factors we assumed to be strictly correlated to the habitat of mosquitoes (average humidity rate and cumulative precipitation, both related to the environmental situation within 15 days prior to mosquito captures) and the mosquito carrying capacity resulted in our study. This indicates that these climatic factors may be not critical determinants of the carrying capacity during the surveillance season (May-November) in the study area.

The inclusion of further environmental factors to the base density-dependent model (Gompertz-logistic) through the IT-AIC_c_ approach allowed definition of the relative importance of each of both density-dependent factors and all of the environmental variables. The length of the daylight, the density-dependent factor included in the Gompertz-logistic model, and the cumulative GDD resulted in being the most important factor variables in describing variation in *Cx. pipiens* per capita growth rates. In contrast, the other environmental predictors considered here (daily GDD, daily precipitation, cumulative precipitation and number of rainy days within 15 days prior to mosquito collections, and minimum daily humidity and average humidity in 15 days before mosquito captures) were judged to have relatively less impact of mosquito population dynamics, on their limited importance as defined through the IT-AIC_c_ approach. We caution that these findings should not be interpreted as dismissing the role of abiotic factors on *Cx. pipiens* dynamics, but as evidence that the upper limit of mosquito population growth rates may be set just as much or more by intra-specific competition than environmental factors.

The IT-AIC_c_ approach used here indicated that a model set with a cumulative *w*AIC_c_ of 0.95, interpreted as being likely to contain the best AIC-model of mosquito population growth, was achieved from the sum weightings of the highest ranked 48 out of the 128 model set. This can be interpreted as reflecting a high level of uncertainty in defining the single best model (with several performing equally well). The relatively similar performance of several alternative models tested here could arise because of correlations between environmental variables, which would consequently limit the amount of additional information derived from varying the specific combinations of variables tested. However, in the model with averaged coefficients, only the three factors with the higher probability to be included in the best AIC-model resulted in having a significant coefficient. The respectively positive and negative sign of the coefficients for the length of daylight and for the density-dependent factors are easily interpretable: *Cx. pipiens* population growth is predicted to go up as day length lengthens, and fall as mosquito density rises. However, interpretation of the negative coefficient for the cumulative growth degree-days in 15 days prior to the capture is less clear. This predicted decrease in population growth rate as the number of growing days during the larval development period increases appears counterintuitive, but may be a product of a non-linear relationship between temperature and growth rate; and/or negative correlations between GDD and other key mosquito resources (e.g. evaporation of larval habitat/reduced rainfall associated with higher GDD). This finding appears to contrast with previous studies on WNV mosquitoes in Italy, which described the population density as positively correlated to the temperature [20,47,48]. As GDD is a composite measure that incorporates not just temperature but the length of time at conditions deemed optimal for growth, it is conceivable that these measures of environmental conditions have differing implications for population growth. Further study is required to investigate this apparent discrepancy in the nature of temperature effects.

While the modelling investigation here has been useful to highlight the contribution of density-dependent as well as environmental factors to *Cx. pipiens* population dynamics; we caution that a substantial amount of variation in mosquito population growth rate remained unexplained by the statistical models tested. Notably, the overall goodness of fit of the 48 models used in model averaging was marginally higher than that of the base population dynamics model (which had a rank of 65 out of the 128 tested). Thus further more detailed analyses including data from a longer time series and/or incorporation of additional environmental variables from what was measured here, and their interactions will be required to obtain a model that can predict *Cx. pipiens* population growth with a high degree of quantitative accuracy; as opposed to revealing the relative strength and direction with which environmental factors and density-dependence influence these dynamics, as done here.

In seeking to estimate the impact of environmental factors in driving this process, a frequently-used approach (and one used here) is to test for the impact of specific environmental factors while controlling for the random effect of site. A possible limitation of this approach is that some environmental factors may be relatively static across time. This could make it difficult to distinguish the relative impact of ‘site’ from ‘site-specific’ environmental factors, and potentially lead to an underestimation of the relative importance of environmental factors considered independently of site. While we acknowledge this as a potential limitation of this approach, we do not feel it could have created a bias in our results as all the environmental variables we considered (rainfall, temperature and humidity-related) varied very extensively within a site over all years, and had little collinearity with ‘site’.

Whilst the statistical models tested here were subject to some limitations, including the assumption of a fixed length of the mosquito life cycle and larval development throughout the study period, our results provide useful insights into the population dynamics of *Cx. pipiens* in Western Europe. Most notably, the inclusion of density-dependence yielded better prediction of their population growth rates. Consequently, failure to account for density dependence when analysing mosquito population growth rate data may lead to biased inference, especially when the population density becomes remarkably high. Mosquito populations may respond in different ways to fluctuations of environmental conditions according to their abundances. Specifically, at the start of the mosquito growing season when numbers are low, small increases in favourable environmental conditions may lead to rapid increase in population growth. In contrast, when mosquito population density is very high and intra-specific competition high, further improvements in environmental conditions may yield no change as population growth is suppressed by competition [21-23,49]. Density-dependent models allow for these intrinsic regulatory effects to be incorporated, and thus can provide information on how populations may respond to environmental change that would be missed in studies that consider only the linear effects of environmental factors.

Although the inclusion of density-dependence as shown here can significantly improve our understanding of mosquito population dynamics, we caution that further investigation is required in a wider range of areas that differ in macro-ecological and socio-economic factors to confirm the generality of this conclusion. Furthermore, it could be that wider consideration of other environmental and demographic data that were not included here could yield significantly better predictions of mosquito and population dynamics, and thus reduce the relative importance of density –dependence from what has been described here. However, density dependence is known to play an important role in regulating animal populations in many other ecological systems [50], and we hypothesize it is likely to remain important in this and other mosquito species even in other environmental conditions; albeit its relative importance over climatic factors may be reduced or reversed in areas with more extreme environmental variations in time.

Mosquito species collected within the 2-year surveillance (2009–2011) conducted here included not only *Cx. pipiens*, but many others that may act as vectors for several VBDs. In addition to WNV, a number of newly introduced arboviruses have been detected in the past five years in Italy. Besides WNV which now appears to be endemic in north-eastern Italy, other viruses including Usutu and Chikungunya viruses have also been isolated from mosquitoes collected in this study, or in adjoining regions [51-53]. These events, together with the introduction of exotic mosquito species that could be related to both climate changes and increased global trade [54], make knowledge of mosquito population dynamics of paramount importance to better understand mechanisms of VBD dissemination and persistence.

## Conclusions

Our study provides a first attempt to gain a deeper insight into WNV vector dynamics in Italy. The inclusion of growth rate fluctuations, and of density dependent effects, could enhance models to predict the trend of mosquito populations in areas considered at risk of WNV introduction and spread. Furthermore, the trend of growth rate could give a better idea on when and where to expect an increase in vector population density, and therefore an amplified risk of WNV transmission. Based on this information, appropriate intervention measures such as the implementation of risk-based surveillance programmes could be envisaged, in order to better assess the risk of human infections.

## Competing interest

The authors declare that they have no competing interests.

## Authors’ contributions

PM conducted the analyses and wrote the manuscript. HMF helped in designing the study, interpreting the results from the models and drafting the manuscript, GC and FM provided the data, supervised the analyses and contributed to the interpretation, LB and SM provided consulting advice and supervised the drafting of the manuscript. All authors read and approved the final version of the manuscript.

## Supplementary Material

Additional file 1: Table S1 Alternative models accounting for the addition of combinations of environmental factors to the baseline population model. The models are reported in order of increasing ΔAICc, the baseline model is reported in boldface; the models that were averaged to obtain a full model are higlighted in the gray-shaded area. Click here for file
