# Supplementary material for: Determinants of the population growth of the West Nile virus mosquito vector Culex pipiens in a repeatedly affected area in Italy
Source: Parasit Vectors. 2014 Jan 15;7:26. doi: 10.1186/1756-3305-7-26 (PMC3896690; doi:10.1186/1756-3305-7-26)
Supplement: Additional file 1: Table S1 — Alternative models accounting for the addition of combinations of environmental factors to the baseline population model. The models are reported in order of increasing ΔAICc, the baseline model is reported in boldface; the models that were averaged to obtain a full model are higlighted in the gray-shaded area. [file 1756-3305-7-26-S1.docx]

| **Table S1. Alternative models accounting for the addition of combinations of environmental factors to the baseline population model.**  The models are reported in order of increasing ΔAICc, the baseline model is reported in boldface; the models that were averaged to obtain a full model are higlighted in the gray-shaded area. | | | | | |
| --- | --- | --- | --- | --- | --- |
| **Rank** | **Model** | **ΔAICc** | ***w*AIC_c_** | **Cumulative *w*AIC_c_** | ***R^2^_LR_*** |
| 1 | Base model+ GDD+ GDD.15d | 0.000 | 0.077 | 0.077 | 0.60 |
| 2 | Base model + DPREC.15d+ GDD+ GDD.15d | 0.489 | 0.061 | 0.138 | 0.60 |
| 3 | Base model + DPREC.15d+ GDD.15d | 0.719 | 0.054 | 0.192 | 0.60 |
| 4 | Base model+ GDD.15d | 0.736 | 0.054 | 0.246 | 0.60 |
| 5 | Base model+ GDD+ GDD.15d+ HAV.15d | 0.970 | 0.048 | 0.294 | 0.60 |
| 6 | Base model+ GDD.15d+ HAV.15d | 1.437 | 0.038 | 0.331 | 0.60 |
| 7 | Base model+ PREC.15d+ GDD+ GDD.15d | 1.860 | 0.031 | 0.362 | 0.60 |
| 8 | Base model+ PREC+ GDD+ GDD.15d | 1.926 | 0.030 | 0.392 | 0.60 |
| 9 | Base model+ GDD+ GDD.15d+ HMN | 2.034 | 0.028 | 0.420 | 0.60 |
| 10 | Base model + DPREC.15d+ PREC+ GDD+ GDD.15d | 2.123 | 0.027 | 0.446 | 0.60 |
| 11 | Base model + DPREC.15d+ PREC.15d+ GDD+ GDD.15d | 2.223 | 0.025 | 0.472 | 0.60 |
| 12 | Base model + DPREC.15d+ PREC.15d+ GDD.15d | 2.405 | 0.023 | 0.495 | 0.60 |
| 13 | Base model + DPREC.15d+ GDD+ GDD.15d+ HAV.15d | 2.411 | 0.023 | 0.518 | 0.60 |
| 14 | Base model + DPREC.15d+ GDD+ GDD.15d+ HMN | 2.468 | 0.023 | 0.541 | 0.60 |
| 15 | Base model+ PREC.15d+ GDD.15d | 2.497 | 0.022 | 0.563 | 0.60 |
| 16 | Base model + DPREC.15d+ GDD.15d+ HAV.15d | 2.635 | 0.021 | 0.584 | 0.60 |
| 17 | Base model+ GDD.15d+ HMN | 2.685 | 0.020 | 0.604 | 0.60 |
| 18 | Base model + DPREC.15d+ PREC+ GDD.15d | 2.698 | 0.020 | 0.624 | 0.60 |
| 19 | Base model+ PREC+ GDD.15d | 2.754 | 0.020 | 0.644 | 0.60 |
| 20 | Base model + DPREC.15d+ GDD.15d+ HMN | 2.755 | 0.020 | 0.663 | 0.60 |
| 21 | Base model+ PREC+ GDD+ GDD.15d+ HAV.15d | 2.826 | 0.019 | 0.682 | 0.60 |
| 22 | Base model+ GDD+ GDD.15d+ HMN+ HAV.15d | 2.955 | 0.018 | 0.700 | 0.60 |
| 23 | Base model+ PREC.15d+ GDD+ GDD.15d+ HAV.15d | 3.006 | 0.017 | 0.717 | 0.60 |
| 24 | Base model+ GDD.15d+ HMN+ HAV.15d | 3.468 | 0.014 | 0.731 | 0.60 |
| 25 | Base model+ PREC.15d+ GDD.15d+ HAV.15d | 3.474 | 0.014 | 0.744 | 0.60 |
| 26 | Base model+ PREC+ GDD.15d+ HAV.15d | 3.475 | 0.014 | 0.758 | 0.60 |
| **Rank** | **Model** | **ΔAICc** | ***w*AIC_c_** | **Cumulative *w*AIC_c_** | **R^2^** |
| 27 | Base model+ PREC+ PREC.15d+ GDD+ GDD.15d | 3.704 | 0.012 | 0.770 | 0.60 |
| 28 | Base model+ PREC.15d+ GDD+ GDD.15d+ HMN | 3.869 | 0.011 | 0.781 | 0.60 |
| 29 | Base model + DPREC.15d+ PREC+ PREC.15d+ GDD+ GDD.15d | 3.928 | 0.011 | 0.792 | 0.60 |
| 30 | Base model+ PREC+ GDD+ GDD.15d+ HMN | 3.957 | 0.011 | 0.803 | 0.60 |
| 31 | Base model + DPREC.15d+ PREC+ GDD+ GDD.15d+ HAV.15d | 4.074 | 0.010 | 0.813 | 0.60 |
| 32 | Base model + DPREC.15d+ PREC.15d+ GDD+ GDD.15d+ HAV.15d | 4.104 | 0.010 | 0.823 | 0.60 |
| 33 | Base model + DPREC.15d+ PREC+ GDD+ GDD.15d+ HMN | 4.166 | 0.010 | 0.833 | 0.60 |
| 34 | Base model + DPREC.15d+ PREC.15d+ GDD+ GDD.15d+ HMN | 4.251 | 0.009 | 0.842 | 0.60 |
| 35 | Base model + DPREC.15d+ PREC.15d+ GDD.15d+ HAV.15d | 4.276 | 0.009 | 0.851 | 0.60 |
| 36 | Base model + DPREC.15d+ GDD+ GDD.15d+ HMN+ HAV.15d | 4.374 | 0.009 | 0.860 | 0.60 |
| 37 | Base model + DPREC.15d+ PREC+ PREC.15d+ GDD.15d | 4.412 | 0.009 | 0.868 | 0.60 |
| 38 | Base model + DPREC.15d+ PREC.15d+ GDD.15d+ HMN | 4.415 | 0.009 | 0.877 | 0.60 |
| 39 | Base model+ PREC.15d+ GDD.15d+ HMN | 4.511 | 0.008 | 0.885 | 0.60 |
| 40 | Base model+ PREC+ PREC.15d+ GDD.15d | 4.535 | 0.008 | 0.893 | 0.60 |
| 41 | Base model + DPREC.15d+ PREC+ GDD.15d+ HAV.15d | 4.627 | 0.008 | 0.901 | 0.60 |
| 42 | Base model + DPREC.15d+ GDD.15d+ HMN+ HAV.15d | 4.676 | 0.007 | 0.908 | 0.60 |
| 43 | Base model + DPREC.15d+ PREC+ GDD.15d+ HMN | 4.709 | 0.007 | 0.916 | 0.60 |
| 44 | Base model+ PREC+ GDD.15d+ HMN | 4.722 | 0.007 | 0.923 | 0.60 |
| 45 | Base model+ PREC+ GDD+ GDD.15d+ HMN+ HAV.15d | 4.867 | 0.007 | 0.930 | 0.60 |
| 46 | Base model+ PREC+ PREC.15d+ GDD+ GDD.15d+ HAV.15d | 4.869 | 0.007 | 0.936 | 0.60 |
| 47 | Base model+ PREC.15d+ GDD+ GDD.15d+ HMN+ HAV.15d | 4.999 | 0.006 | 0.943 | 0.60 |
| 48 | Base model+ PREC.15d+ GDD.15d+ HMN+ HAV.15d | 5.507 | 0.005 | 0.948 | 0.60 |
| 49 | Base model+ PREC+ GDD.15d+ HMN+ HAV.15d | 5.507 | 0.005 | 0.953 | 0.60 |
| 50 | Base model+ PREC+ PREC.15d+ GDD.15d+ HAV.15d | 5.515 | 0.005 | 0.958 | 0.60 |
| 51 | Base model+ PREC+ PREC.15d+ GDD+ GDD.15d+ HMN | 5.748 | 0.004 | 0.962 | 0.60 |
| 52 | Base model + DPREC.15d+ PREC+ PREC.15d+ GDD+ GDD.15d+ HAV.15d | 5.845 | 0.004 | 0.966 | 0.60 |
| 53 | Base model + DPREC.15d+ PREC+ PREC.15d+ GDD+ GDD.15d+ HMN | 5.960 | 0.004 | 0.970 | 0.60 |
| 54 | Base model + DPREC.15d+ PREC+ GDD+ GDD.15d+ HMN+ HAV.15d | 6.120 | 0.004 | 0.974 | 0.60 |
| 55 | Base model + DPREC.15d+ PREC.15d+ GDD+ GDD.15d+ HMN+ HAV.15d | 6.125 | 0.004 | 0.977 | 0.60 |
| 56 | Base model + DPREC.15d+ PREC+ PREC.15d+ GDD.15d+ HAV.15d | 6.296 | 0.003 | 0.981 | 0.60 |
| **Rank** | **Model** | **ΔAICc** | ***w*AIC_c_** | **Cumulative *w*AIC_c_** | **R^2^** |
| 57 | Base model + DPREC.15d+ PREC.15d+ GDD.15d+ HMN+ HAV.15d | 6.300 | 0.003 | 0.984 | 0.60 |
| 58 | Base model + DPREC.15d+ PREC+ PREC.15d+ GDD.15d+ HMN | 6.373 | 0.003 | 0.987 | 0.60 |
| 59 | Base model+ PREC+ PREC.15d+ GDD.15d+ HMN | 6.546 | 0.003 | 0.990 | 0.60 |
| 60 | Base model + DPREC.15d+ PREC+ GDD.15d+ HMN+ HAV.15d | 6.655 | 0.003 | 0.993 | 0.60 |
| 61 | Base model+ PREC+ PREC.15d+ GDD+ GDD.15d+ HMN+ HAV.15d | 6.913 | 0.002 | 0.995 | 0.60 |
| 62 | Base model+ PREC+ PREC.15d+ GDD.15d+ HMN+ HAV.15d | 7.549 | 0.002 | 0.997 | 0.60 |
| 63 | Base model + DPREC.15d+ PREC+ PREC.15d+ GDD+ GDD.15d+ HMN+ HAV.15d | 7.888 | 0.002 | 0.999 | 0.60 |
| 64 | Base model + DPREC.15d+ PREC+ PREC.15d+ GDD.15d+ HMN+ HAV.15d | 8.285 | 0.001 | 1.000 | 0.60 |
| 65 | Base model | 17.331 | 0.000 | 1.000 | 0.59 |
| 66 | Base model+ PREC.15d | 17.481 | 0.000 | 1.000 | 0.59 |
| 67 | Base model + DPREC.15d | 18.369 | 0.000 | 1.000 | 0.59 |
| 68 | Base model++ HAV.15d | 18.814 | 0.000 | 1.000 | 0.59 |
| 69 | Base model+ PREC+ PREC.15d | 19.052 | 0.000 | 1.000 | 0.59 |
| 70 | Base model+ PREC | 19.189 | 0.000 | 1.000 | 0.59 |
| 71 | Base model+ HMN | 19.232 | 0.000 | 1.000 | 0.59 |
| 72 | Base model+ PREC.15d+ GDD | 19.334 | 0.000 | 1.000 | 0.59 |
| 73 | Base model+ GDD | 19.343 | 0.000 | 1.000 | 0.59 |
| 74 | Base model+ PREC.15d+ HAV.15d | 19.512 | 0.000 | 1.000 | 0.59 |
| 75 | Base model+ PREC.15d+ HMN | 19.512 | 0.000 | 1.000 | 0.59 |
| 76 | Base model + DPREC.15d+ PREC.15d | 19.514 | 0.000 | 1.000 | 0.59 |
| 77 | Base model + DPREC.15d+ PREC | 20.038 | 0.000 | 1.000 | 0.59 |
| 78 | Base model + DPREC.15d+ GDD | 20.216 | 0.000 | 1.000 | 0.59 |
| 79 | Base model + DPREC.15d++ HMN | 20.380 | 0.000 | 1.000 | 0.59 |
| 80 | Base model + DPREC.15d++ HAV.15d | 20.400 | 0.000 | 1.000 | 0.59 |
| 81 | Base model+ PREC++ HAV.15d | 20.627 | 0.000 | 1.000 | 0.59 |
| 82 | Base model+ GDD+ HAV.15d | 20.756 | 0.000 | 1.000 | 0.59 |
| 83 | Base model++ HMN+ HAV.15d | 20.800 | 0.000 | 1.000 | 0.59 |
| 84 | Base model+ PREC+ HMN | 20.837 | 0.000 | 1.000 | 0.59 |
| 85 | Base model+ PREC+ PREC.15d+ GDD | 21.001 | 0.000 | 1.000 | 0.59 |
| 86 | Base model+ PREC+ PREC.15d+ HMN | 21.019 | 0.000 | 1.000 | 0.59 |
| **Rank** | **Model** | **ΔAICc** | ***w*AIC_c_** | **Cumulative *w*AIC_c_** | **R^2^** |
| 87 | Base model+ PREC+ PREC.15d+ HAV.15d | 21.081 | 0.000 | 1.000 | 0.59 |
| 88 | Base model + DPREC.15d+ PREC+ PREC.15d | 21.089 | 0.000 | 1.000 | 0.59 |
| 89 | Base model+ GDD+ HMN | 21.212 | 0.000 | 1.000 | 0.59 |
| 90 | Base model+ PREC+ GDD | 21.221 | 0.000 | 1.000 | 0.59 |
| 91 | Base model + DPREC.15d+ PREC.15d+ GDD | 21.369 | 0.000 | 1.000 | 0.59 |
| 92 | Base model+ PREC.15d+ GDD+ HMN | 21.371 | 0.000 | 1.000 | 0.59 |
| 93 | Base model+ PREC.15d+ GDD+ HAV.15d | 21.372 | 0.000 | 1.000 | 0.59 |
| 94 | Base model+ PREC.15d+ HMN+ HAV.15d | 21.547 | 0.000 | 1.000 | 0.59 |
| 95 | Base model + DPREC.15d+ PREC.15d+ HMN | 21.549 | 0.000 | 1.000 | 0.59 |
| 96 | Base model + DPREC.15d+ PREC.15d+ HAV.15d | 21.549 | 0.000 | 1.000 | 0.59 |
| 97 | Base model + DPREC.15d+ PREC+ HMN | 21.861 | 0.000 | 1.000 | 0.59 |
| 98 | Base model + DPREC.15d+ PREC+ GDD | 21.969 | 0.000 | 1.000 | 0.59 |
| 99 | Base model + DPREC.15d+ PREC++ HAV.15d | 22.075 | 0.000 | 1.000 | 0.59 |
| 100 | Base model + DPREC.15d+ GDD+ HMN | 22.195 | 0.000 | 1.000 | 0.59 |
| 101 | Base model + DPREC.15d+ GDD+ HAV.15d | 22.249 | 0.000 | 1.000 | 0.59 |
| 102 | Base model + DPREC.15d++ HMN+ HAV.15d | 22.416 | 0.000 | 1.000 | 0.59 |
| 103 | Base model+ PREC+ HMN+ HAV.15d | 22.430 | 0.000 | 1.000 | 0.59 |
| 104 | Base model+ PREC+ GDD+ HAV.15d | 22.622 | 0.000 | 1.000 | 0.59 |
| 105 | Base model+ GDD+ HMN+ HAV.15d | 22.710 | 0.000 | 1.000 | 0.59 |
| 106 | Base model+ PREC+ GDD+ HMN | 22.853 | 0.000 | 1.000 | 0.59 |
| 107 | Base model+ PREC+ PREC.15d+ GDD+ HMN | 22.950 | 0.000 | 1.000 | 0.59 |
| 108 | Base model + DPREC.15d+ PREC+ PREC.15d+ GDD | 23.036 | 0.000 | 1.000 | 0.59 |
| 109 | Base model+ PREC+ PREC.15d+ GDD+ HAV.15d | 23.039 | 0.000 | 1.000 | 0.59 |
| 110 | Base model+ PREC+ PREC.15d+ HMN+ HAV.15d | 23.049 | 0.000 | 1.000 | 0.59 |
| 111 | Base model + DPREC.15d+ PREC+ PREC.15d+ HMN | 23.059 | 0.000 | 1.000 | 0.59 |
| 112 | Base model + DPREC.15d+ PREC+ PREC.15d+ HAV.15d | 23.116 | 0.000 | 1.000 | 0.59 |
| 113 | Base model + DPREC.15d+ PREC.15d+ GDD+ HAV.15d | 23.407 | 0.000 | 1.000 | 0.59 |
| 114 | Base model + DPREC.15d+ PREC.15d+ GDD+ HMN | 23.408 | 0.000 | 1.000 | 0.59 |
| 115 | Base model+ PREC.15d+ GDD+ HMN+ HAV.15d | 23.412 | 0.000 | 1.000 | 0.59 |
| 116 | Base model + DPREC.15d+ PREC.15d+ HMN+ HAV.15d | 23.587 | 0.000 | 1.000 | 0.59 |
| **Rank** | **Model** | **ΔAICc** | ***w*AIC_c_** | **Cumulative *w*AIC_c_** | **R^2^** |
| 117 | Base model + DPREC.15d+ PREC+ GDD+ HMN | 23.751 | 0.000 | 1.000 | 0.59 |
| 118 | Base model + DPREC.15d+ PREC+ HMN+ HAV.15d | 23.900 | 0.000 | 1.000 | 0.59 |
| 119 | Base model + DPREC.15d+ PREC+ GDD+ HAV.15d | 24.009 | 0.000 | 1.000 | 0.59 |
| 120 | Base model + DPREC.15d+ GDD+ HMN+ HAV.15d | 24.234 | 0.000 | 1.000 | 0.59 |
| 121 | Base model+ PREC+ GDD+ HMN+ HAV.15d | 24.397 | 0.000 | 1.000 | 0.59 |
| 122 | Base model + DPREC.15d+ PREC+ PREC.15d+ GDD+ HMN | 24.985 | 0.000 | 1.000 | 0.59 |
| 123 | Base model+ PREC+ PREC.15d+ GDD+ HMN+ HAV.15d | 24.990 | 0.000 | 1.000 | 0.59 |
| 124 | Base model + DPREC.15d+ PREC+ PREC.15d+ GDD+ HAV.15d | 25.069 | 0.000 | 1.000 | 0.59 |
| 125 | Base model + DPREC.15d+ PREC+ PREC.15d+ HMN+ HAV.15d | 25.083 | 0.000 | 1.000 | 0.59 |
| 126 | Base model + DPREC.15d+ PREC.15d+ GDD+ HMN+ HAV.15d | 25.450 | 0.000 | 1.000 | 0.59 |
| 127 | Base model + DPREC.15d+ PREC+ GDD+ HMN+ HAV.15d | 25.794 | 0.000 | 1.000 | 0.59 |
| 128 | Base model + DPREC.15d+ PREC+ PREC.15d+ GDD+ HMN+ HAV.15d | 27.014 | 0.000 | 1.000 | 0.59 |
